# Optimization of the Enrichment of Circulating Tumor Cells for Downstream Phenotypic Analysis in Patients with Non-Small Cell Lung Cancer Treated with Anti-PD-1 Immunotherapy

**DOI:** 10.3390/cancers12061556

**Published:** 2020-06-12

**Authors:** Maria A Papadaki, Afroditi I Sotiriou, Christina Vasilopoulou, Maria Filika, Despoina Aggouraki, Panormitis G Tsoulfas, Christina A Apostolopoulou, Konstantinos Rounis, Dimitrios Mavroudis, Sofia Agelaki

**Affiliations:** 1 Laboratory of Translational Oncology, School of Medicine, University of Crete, 71110 Heraklion, Vassilika Vouton, Crete, Greece; papadaki_maria1@yahoo.gr (M.A.P.); gc250@stud.uni-heidelberg.de (A.I.S.); bio2666@edu.biology.uoc.gr (C.V.); bio2064@edu.biology.uoc.gr (M.F.); daggouraki@yahoo.co.uk (D.A.); bio2241@edu.biology.uoc.gr (P.G.T.); christinapostol@yahoo.gr (C.A.A.); mavroudis@uoc.gr (D.M.); 2 Department of Medical Oncology, University General Hospital of Heraklion, 71110 Heraklion, Vassilika Vouton, Crete, Greece; rounis@gmail.com

**Keywords:** non-small cell lung cancer, NSCLC, immunotherapy, immune checkpoint inhibitors, ICIs, programmed cell death-1 ligand, PD-L1, indoleamine-2,3-dioxygenase, IDO, immune evasion, liquid biopsy, circulating tumor cells, CTCs, ISET, Parsortix

## Abstract

The current study aimed at the optimization of circulating tumor cell (CTC) enrichment for downstream protein expression analyses in non-small cell lung cancer (NSCLC) to serve as a tool for the investigation of immune checkpoints in real time. Different enrichment approaches—ficoll density, erythrolysis, their combination with magnetic separation, ISET, and Parsortix—were compared in spiking experiments using the A549, H1975, and SKMES-1 NSCLC cell lines. The most efficient methods were tested in patients (*n* = 15) receiving immunotherapy targeting programmed cell death-1 (PD-1). Samples were immunofluorescently stained for a) cytokeratins (CK)/epithelial cell adhesion molecule (EpCAM)/leukocyte common antigen (CD45), and b) CK/programmed cell death ligand-1 (PD-L1)/ indoleamine-2,3-dioxygenase (IDO). Ficoll, ISET, and Parsortix presented the highest yields and compatibility with phenotypic analysis; however, at the patient level, they provided discordant CTC positivity (13%, 33%, and 60% of patients, respectively) and enriched for distinct CTC populations. IDO and PD-L1 were expressed in 44% and 33% and co-expressed in 19% of CTCs. CTC detection was associated with progressive disease (PD) (*p* = 0.006), reduced progression-free survival PFS (*p* = 0.007), and increased risk of relapse (hazard ratio; HR: 10.733; *p* = 0.026). IDO-positive CTCs were associated with shorter PFS (*p* = 0.039) and overall survival OS (*p* = 0.021) and increased risk of death (HR: 5.462; *p* = 0.039). The current study indicates that CTC analysis according to distinct immune checkpoints is feasible and may provide valuable biomarkers to monitor NSCLC patients treated with anti-PD-1 agents.

## 1. Introduction

Lung cancer is the most commonly diagnosed cancer and the leading cause of cancer-related deaths for both men and women worldwide [1]. Non-small cell lung cancer (NSCLC), accounting for 84% of all lung cancer cases, is often diagnosed at an advanced or metastatic stage with dismal 5-year survival rates [2]. During the last years, immunotherapy with immune checkpoint inhibitors (ICIs) targeting programmed cell death-1 (PD-1) or programmed cell death ligand-1 (PD-L1) has revolutionized the treatment of advanced NSCLC [3,4]. Currently, ICIs represent a standard treatment for the majority of NSCLC patients. However, significant benefit is provided in only a subset of patients [5], whereas acquired resistance is common among those who initially responded [6].

PD-L1 expression represents a key molecule of immune escape and the only approved predictive biomarker used in clinical practice to inform on the use of anti-PD-1 agents in NSCLC [7]. However, other immune checkpoints involved in the tumor immune surveillance may also affect immunotherapy outcomes. Indoleamine-2,3-dioxygenase (IDO) has been shown to promote tumor evasion from both the innate and adaptive immune response and to be associated with resistance to anti-PD-1 treatment [8,9,10]. Preclinical findings and evidence from NSCLC tissue suggest that PD-L1 and IDO represent distinct, nonoverlapping routes to immune evasion and highlight their combined targeting using anti-PD-1/PD-L1 and anti-IDO inhibitors as a promising therapeutic strategy [11,12,13,14].

However, tissue analysis is challenged by the lack of tissue availability, tumor heterogeneity, and the dynamic nature of immune response [15,16]. Given these restrains, the liquid biopsy approach is being increasingly investigated as a source for biomarker discovery in NSCLC [17,18,19]. The detection of circulating tumor cells (CTCs) in the peripheral blood of patients with solid tumors represents a promising diagnostic, prognostic, and predictive biomarker in various cancer types [20,21]. The analysis of CTCs is technically challenging due to their significant heterogeneity and rarity. Typically, prior to CTC detection, the sample is enriched for the presence of CTCs using different procedures based on properties that discriminate CTCs from normal blood cells, such as physical characteristics, e.g., size, density, deformability, electrical charge, etc., and/or marker expression. A multitude of sensitive and specific CTC enrichment technologies have been introduced over the past decade; however, different methods provide discordant positivity rates and CTC counts even when the same patient sample is being analyzed [22,23,24,25].

In NSCLC, CTC analysis is highly demanding due to low CTC-positivity rates compared to other cancers [26,27,28]. On the other hand, accumulating evidence supports the clinical relevance of CTC detection in NSCLC [26,29,30,31]. Moreover, recent reports highlighted the significant heterogeneity of CTCs in patients with lung cancer [32]. In NSCLC, CTC analysis has mainly focused on the identification of targetable molecular alterations [22,33,34,35]. However, further characterization of the protein expression profile of single CTCs may provide additional prognostic information and could significantly contribute to the understanding of mechanisms of disease progression and resistance to current therapies [36,37,38,39,40].

The lack of standardized procedures for CTC isolation and characterization restricts their application as biomarkers in daily practice [41]. In addition, the genotypic and phenotypic analysis of CTCs may confer differential results according to the methodology used for their enrichment. Therefore, there is a highly unmet need for further validation and optimization of CTC enrichment and detection methods and for the selection of the optimal approach according to the type of intended analysis [42].

In the current report, we aimed at the optimization of CTC isolation for downstream protein expression analyses in patients with NSCLC. To this end, we initially compared the analytical performance of various enrichment approaches in spiking experiments using representative NSCLC cell lines. We tested the most commonly used manual methods, namely, ficoll density gradient centrifugation, red blood cell lysis, and the combination of each one with negative magnetic separation using CD45-coated beads [43,44]. We also tested the efficacy of two automated systems, the size-based filtration platform, ISET [29], and the size- and deformability-based microfluidics system, Parsortix [45]. The performance of the most efficient methods was subsequently evaluated in parallel in a small cohort of patients with NSCLC treated with anti-PD-1 agents, and CTC characterization according to the expression of PD-L1 and IDO was developed and optimized.

We found that ficoll density gradient, ΙSET, and Parsortix provided the highest yield and compatibility for protein expression analysis in spiking experiments. However, when tested in patient samples, they resulted in discordant CTC-positivity rates and enriched for distinct CTC populations. CTC detection by either Parsortix or ISET was associated with poor patient outcome; however, prognostic value was significantly improved when positivity was defined according to the detection by any of the three methods. The current study demonstrates for the first time that IDO is expressed on CTCs and suggests that IDO+ CTCs and particularly the IDO+/PD-L1- subset may have prognostic relevance in patients with NSCLC treated with anti-PD-1 immunotherapy. Although these findings derive from the evaluation of a small cohort of patients, they indicate that the parallel assessment of distinct immune checkpoints on CTCs is feasible in NSCLC and may provide significant prognostic information for the improvement of risk stratification of patients with NSCLC treated with ICIs.

## 2. Results

### 2.1. Optimization of Cell Harvest on Glass Slides for Downstream Immunofluorescence Analysis

**Figure 1:**
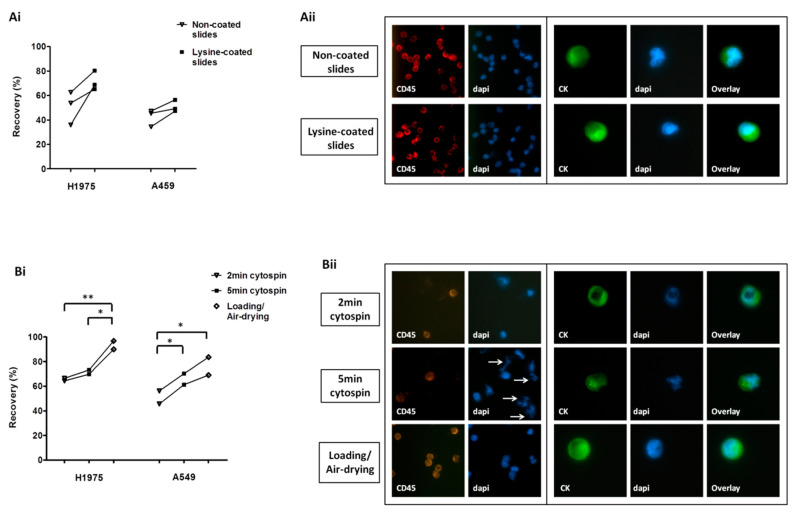
Optimization of cell harvest on slides for downstream protein expression analysis: (**Ai**) Recovery of pre-labeled H1975 and A549 spiked cells transferred on lysine-non-coated and lysine-coated SuperFrost Plus™ adhesion slides. (**Aii**) Immunofluorescence staining among lysine-non-coated and lysine-coated slides; representative staining of leukocyte common antigen (CD45)/DAPI on peripheral blood mononuclear cells (PBMCs) and of cytokeratins (CK)/DAPI on non-pre-labeled A549 cells (Ariol microscopy system, Genetix, New Milton, UK) (400×). (**Bi**) Recovery of pre-labeled cells using different cell harvest approaches. *^,^ ** One-way analysis of variance (ANOVA) test; statistical significance at the *p* < 0.05 level. (**Bii**) Representative CD45/DAPI staining on PBMCs and CK/DAPI staining on non-pre-labeled A549 cells (Ariol microscopy system, 400×) following their transfer on lysine-coated slides with different approaches. Nuclear fragmentation indicated by arrows.

H1975 and A549 cell lines were used in spiking experiments to compare different approaches for cell transfer on glass slides. The use of lysine-coated SuperFrost Plus™ slides (Thermo Fisher Scientific, Waltham, MA, USA) was associated with increased yield for both cell lines compared to non-lysine coated slides (mean recovery; H1975: 71.4% and 50.8%, respectively; A549: 50.9% and 42.4%, respectively) (Figure 1Ai). There was no difference in the morphology and the staining of cells among non-coated and lysine-coated slides (Figure 1Aii). ImageJ (National Institutes of Health (NIH) Bethesda, MD, USA) analysis also revealed that lysine-coating had no effect on the nuclear area and roundness of cells or the intensity of the fluorescent signal (Figure S1Ai–iii).

Among the different harvest approaches tested—cytospin at 500 × *g* for 2 min, cytospin at 500 × *g* for 5 min, and direct loading/air-drying—the last one provided the highest recovery rates for both cell lines (mean recovery; H1975: 65.4%, 71.5%, and 93.4%, respectively; A549: 50.9%, 65.7%, and 76.3%, respectively) (Figure 1Bi). Following 5-min cytospin, several fragments were observed, which were positive for DAPI but negative for CK and CD45 and might represent nuclear fragmentation (Figure 1Bii). ImageJ analysis revealed no difference in the nuclear area or roundness among differentially processed cells; however a significantly reduced staining intensity for CD45 was recorded after 5 min of cytospinning compared to the other methods (Figure S1Bi–iii).

### 2.2. Comparison of Different CTC-Enrichment Methods for Downstream Immunofluorescence Analysis

**Figure 2:**
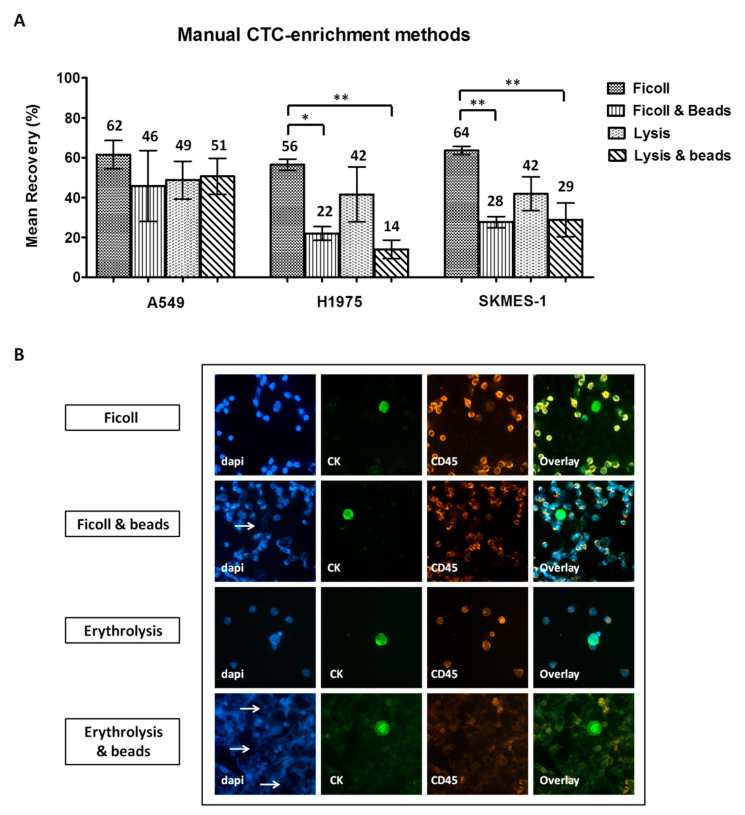
Comparison of manual circulating tumor cell (CTC)-enrichment approaches in spiking experiments: (**A**) Recovery rates of pre-labeled A549, H1975, and SKMES-1 cells following enrichment with each method. Values represent mean recoveries; error bars represent standard error of mean (SEM). *^,^ ** One-way ANOVA test; statistical significance at the *p* < 0.001 level. (**B**) Representative staining of DAPI/CK/CD45 on spiked non-pre-labeled H1975 cells enriched using different methods (Ariol microscopy system, Genetix, New Milton, UK) (200×). Nuclear fragmentation indicated by arrows.

Three NSCLC cell lines, A549, H1975, and SKMES-1, were used in spiking experiments to compare different enrichment methodologies. The following mean recovery rates (±standard error of mean, SEM) were recorded after enrichment using ficoll, ficoll/beads, erythrolysis, and erythrolysis/beads: a) A549 cells: 62% ± 7%, 46% ± 18%, 49% ± 10%, and 51% ± 9%; b) H1975: 56% ± 3%, 22% ± 3%, 42% ± 14%, and 14% ± 5%; and c) SKMES-1: 64% ± 2%, 28% ± 3%, 42% ± 9%, and 29% ± 8%, respectively (Figure 2A). One-way analysis of variance (ANOVA) test revealed significantly higher recovery rates for H1975 and SKMES-1 cells by using ficoll alone compared to ficoll/beads and to erythrolysis/beads (Tukey’s Multiple Comparison Test, *p* < 0.001). Moreover, numerous DAPI-stained fragments were observed among samples processed by magnetic bead separation, either combined with ficoll and especially with erythrolysis (Figure 2B). In accordance, ImageJ analysis revealed a significantly decreased nuclear area and reduced CD45 staining intensity in these samples (Figure S1Ci–iii).

**Figure 3:**
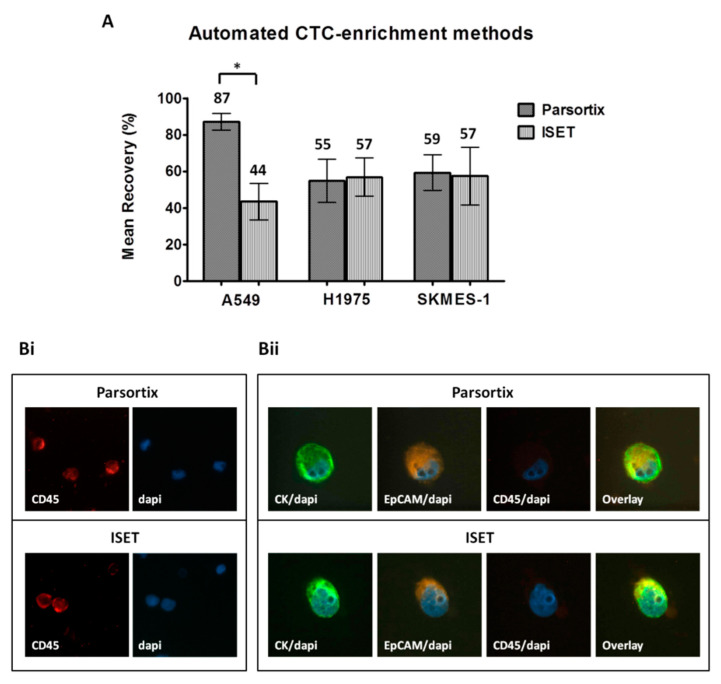
Comparison of the automated CTC-enrichment systems Parsortix and ISET in spiking experiments: Three representative non-small cell lung cancer (NSCLC) cell lines, A549, H1975, and SKMES-1, were used. (**A**) Recovery rates following enrichment by using Parsortix and ISET. Values represent mean recoveries; ± SEM (error bars). * Two-sided t-test; statistical significance observed at the *p* < 0.05 level. (**B**) Representative staining of DAPI/CD45 staining on PBMC (i) and of DAPI/CK/epithelial cell adhesion molecule EpCAM/CD45 staining on spiked H1975 cells (ii) enriched by the two methods (Ariol microscopy system, Genetix, New Milton, UK) (400×).

When comparing the automated approaches, the ISET and Parsortix systems provided similar yield for H1975 cells (55% ± 12% and 57% ± 11%, respectively) and SKMES-1 cells (59% ± 10% and 57% ± 16%, respectively) (Figure 3A). However, higher recovery rates were observed for A549 cells by the use of Parsortix compared to ISET (87% ± 5% and 44% ± 10%, respectively, Two-sided T-test; *p* = 0.017). Both systems resulted in an apparently proper cell morphology and similar immunofluorescence staining intensity (Figure 3Bi,ii). Although ImageJ analysis confirmed that there was no difference in the staining intensity, it revealed a slightly increased nuclear area among cells enriched by ISET, which might however be explained by the bigger size of the ISET membrane pores compared to that of Parsortix cassette gaps (8 μm and 6.5 μm, respectively) (Figure S1Di–iii). Moreover, the occasional localization of cells on a pore of the ISET membranes could complicate the evaluation of intensity and subcellular localization of the markers in the respective cells (Figure S2). 

The throughput of manual and automated enrichment approaches was also compared considering the time required for both the enrichment and subsequent analysis steps. Samples enriched by ficoll or erythrolysis are contaminated with millions of blood cells, resulting in a higher number of slides for evaluation (Table S1). The combination of these methods with negative magnetic separation is time consuming; however it reduces contamination with blood cells and the number of slides for further analysis (mean number of slides per 5 mL blood processed by ficol, ficoll/beads, erythrolysis, and erythrolysis/beads: *n* = 9, 1, 14, and 2, respectively). Blood filtration using the ISET platform is rapid and provides samples of high purity; however, cells were captured in 10 different spots, which were individually stained and analyzed after immobilization on 10 slides. Finally, samples enriched using the Parsortix system are of high purity, and isolated cells can be harvested and transferred in one single slide; however, the enrichment procedure is time consuming (Table S1).

### 2.3. Detection and Characterization of CTCs Enriched by Different Approaches in Patients with NSCLC

#### 2.3.1. Frequency of CTC Detection Following Enrichment Using Different Approaches

CTCs were enriched from peripheral blood samples obtained from 15 patients with NSCLC using ficoll density gradient centrifugation and the ISET and Parsortix systems. Samples were individually stained for CK/EpCAM/CD45 and CK/IDO/PD-L1; the expression of CK and/or EpCAM was used as a marker for CTC identification. CTCs were detected in 13%, 33%, and 60% of patients using ficoll density gradient centrifugation, ISET, and Parsortix, respectively, and in 73% of patients by any of the three methods (Table 1). Four patients harbored CTCs detected by Parsortix only, and two patients harbored CTCs detected by ISET only. In addition, one cluster of 10 CTCs was identified using Parsortix in a patient who also had single CTCs. In the same patient, no CTCs were detected with ficoll or ISET.

As shown in Table 1, Parsortix provided increased CTC yield compared to ficoll and ISET (Friedman exact paired test; *p* = 0.010). When the different methods were compared in pairs, higher CTC counts were detected using Parsortix compared to ficoll (Wilcoxon t-test; *p* = 0.017), whereas no significant difference was shown between Parsortix and ISET (*p* = 0.052) or between ficoll and ISET (*p* = 0.276).

It should be mentioned however that, in 1 out of 15 patients, blood filtration through the ISET membrane was discontinued due to blood clotting, thus resulting in the evaluation of only a portion of the patient sample (4 mL out of 10 mL). In this patient, no CTCs were detected using either ISET or ficoll separation, whereas 2 CTCs were detected following Parsortix enrichment.

#### 2.3.2. Phenotype of CTCs Enriched Using Different Approaches

**Figure 4:**
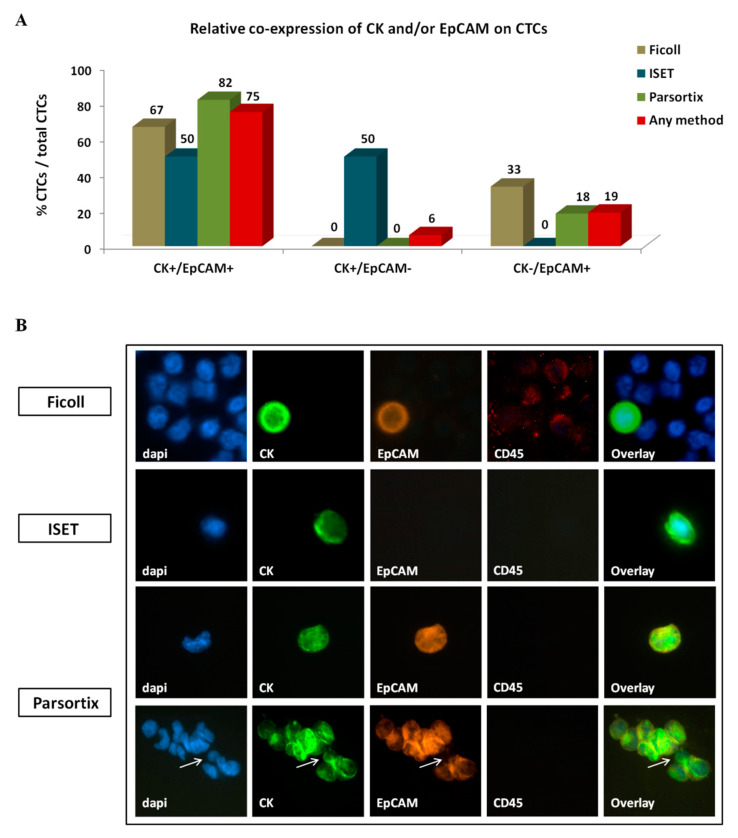
Co-expression of epithelial markers CK and EpCAM on circulating tumor cells (CTCs) of patients with NSCLC: (**A**) Frequency of distinct co-expression phenotypes among CTCs identified using different enrichment approaches. Any method depicts the percentage of a specific phenotype among the total number of CTCs detected by all methods. (**B**) Representative images of CTCs enriched by different methods: All depicted cells co-express CK+/EpCAM+, except the ISET-enriched cell which is of the CK+/EpCAM- phenotype. A CTC cluster identified by Parsortix is also depicted, expressing CK+/EpCAM+ in all except one cell, which is of the CK+/EpCAM− phenotype (arrow). Ariol microscopy system, Genetix, New Milton, UK) (400×).

Three distinct CTC phenotypes were identified according to CK/EpCAM/CD45 staining; one co-expressing CK and EpCAM (CK+/EpCAM+), one expressing CK only (CK+/EpCAM−), and one expressing EpCAM only (CK-/EpCAM+). CD45 expression was not detected in any CK+ or EpCAM+ cell. Interestingly, the distribution of distinct phenotypes varied among CTCs detected using different enrichment methods (Figure 4A,B). CK+/EpCAM+ CTCs constituted the most prevalent CTC population, representing 67%, 50%, and 82% of total CTCs enriched using ficoll, ISET, and Parsortix, respectively (Figure 4A,B). In contrast, CK+/EpCAM− CTCs were identified by ISET only and represented 50% of CTCs detected by this system. CK−/EpCAM+ CTCs were detected by ficoll and Parsortix only, representing 33% and 18% of total CTCs detected by each method, respectively (Figure 4A).

Ten CTCs were detected in the single CTC cluster identified, all of which had the CK+/EpCAM+ phenotype except for one cell bearing the CK+/EpCAM− phenotype (Figure 4B).

#### 2.3.3. Investigation of Immune Checkpoints on CTCs Enriched by Different Approaches

**Figure 5:**
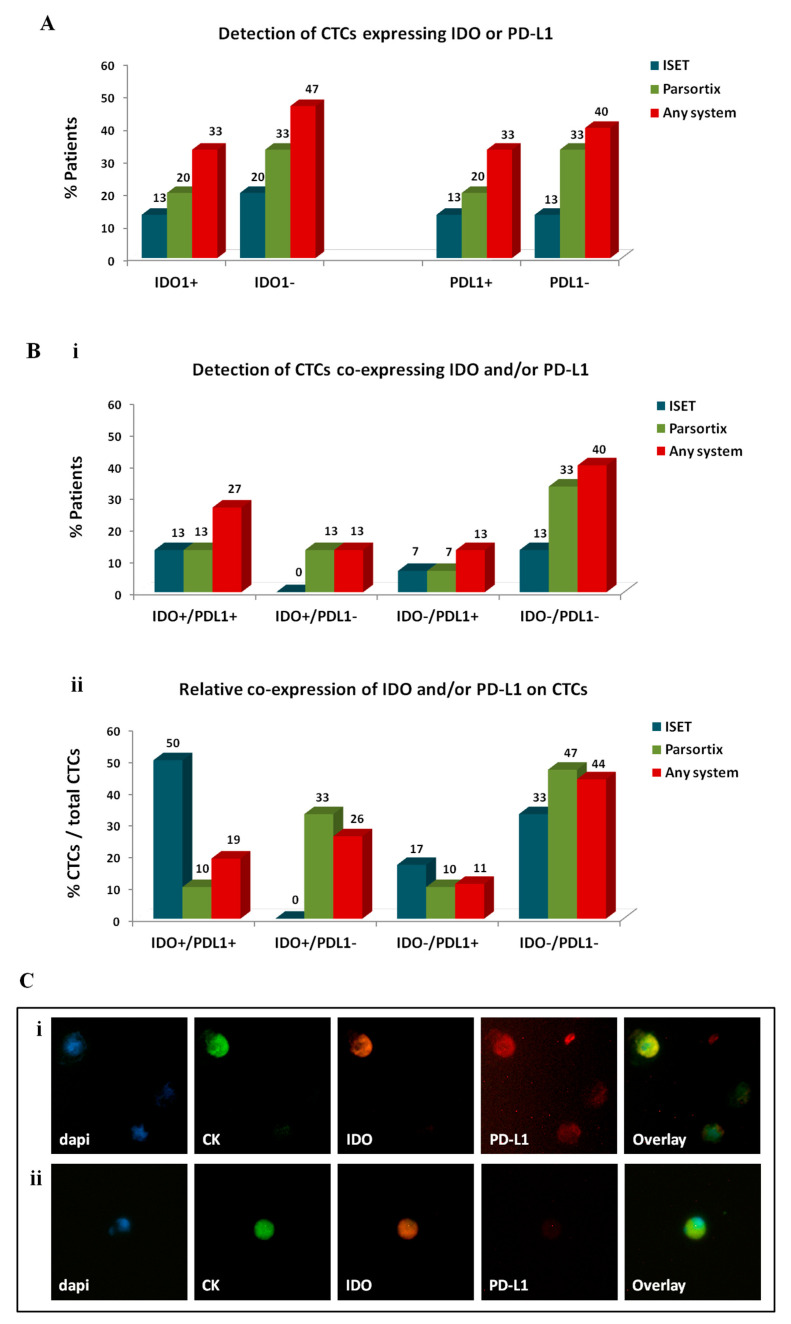
Expression of immune checkpoints, indoleamine-2,3-dioxygenase (IDO) and programmed cell death ligand-1 (PD-L1), on circulating tumor cells (CTCs) of patients with non-small cell lung cancer (NSCLC): Distribution of distinct CTC subsets enriched by different approaches. (**A**) Percentage of patients harbouring CTCs of distinct IDO or PD-L1 phenotypes and (**Bi**) percentage of patients harbouring CTCs of distinct IDO/PD-L1 co-expressing phenotypes. The detection of at least 1 CTC of a specific phenotype was used to define positivity for the respective phenotype. (**Bii**) Percentage of CTCs presenting distinct IDO/PD-L1 co-expressing phenotypes. Any method depicts the percentage of a specific phenotype among the total number of CTCs detected by all methods. Representative images of (**Ci**) IDO+/PD-L1+ CTC and (**Cii**) IDO+/PD-L1− CTC, Ariol microscopy system, Genetix, New Milton, UK) (400×).

To investigate the expression of IDO and PD-L1 on CTCs, samples enriched by different approaches were stained for CK/IDO/PD-L1. CTCs were not detected in ficoll-enriched samples using this staining; therefore, IDO and PD-L1 expression was evaluated on CTCs identified by ISET and Parsortix only (Figure 5).

IDO+ CTCs were identified in 13% of patients by ISET, in 20% by Parsortix, and in 33% by any system (Figure 5A). IDO+ CTCs represented 44% of total CTCs detected by any method, 50% of CTCs detected by ISET, and 43% of CTCs detected by Parsortix.

PD-L1+ CTCs were detected in 13%, 20%, and 33% of patients by ISET, Parsortix, and any system, respectively. PD-L1+ CTCs constituted 30% of total CTCs detected by any method and were more frequently identified using ISET compared to Parsortix (67% and 19% of CTCs detected by each method, respectively).

The distribution of IDO and PD-L1 co-expression on CTCs also varied among the two automated systems (Figure 5B). IDO+/PD-L1+ CTCs were detected in 13% of patients by either ISET or Parsortix; however, their relative percentage among the total CTCs detected by each method was higher in the population detected by ISET compared to Parsortix (50% and 10%, respectively). IDO−/PD-L1− CTCs constituted the most frequent subset enriched by Parsortix (in 33% of patients and in 47% of CTCs) and the second more frequent subset using ISET (in 13% of patients and in 33% of CTCs). Of note, IDO+/PD-L1− CTCs were detected only by Parsortix (in 13% of patients and in 33% of CTCs). Finally, IDO-/PD-L1+ CTCs were evident in 13% of patients by any system and represented 11% of total CTCs. Representative images of IDO+/PD-L1+ CTCs and IDO+/PD-L1- CTCs are depicted in Figure 5C.

### 2.4. Clinical Relevance of CTCs and Distinct CTC Subsets among Patients with Metastatic NSCLC Treated with Anti-PD-1 Inhibitors

#### 2.4.1. Patients

Patient and disease characteristics are summarized in Table 2. At the time of analysis, 13 out of 15 patients had relapsed (median progression-free survival; PFS: 2.8 months (0.1–6.7)), and 9 patients had died (median overall survival; OS: 6.7 months (2.9–10.6)). 

#### 2.4.2. Correlation of CTCs with Clinicopathological Parameters and Response to Anti-PD-1 Inhibitors

The detection of CTCs or of distinct CTC subpopulations was not correlated with age, gender, smoking status, performance status, histology subtype, the number of organs affected, the site of metastases, or the neutrophil-lymphocyte ratio (NLR).

CTCs were detected by any method in 0%, 66.7%, and 100% of patients experiencing partial response (PR), stable disease (SD), and progressive disease (PD), respectively (p = 0.006) (Table 3). Only CTCs detected by Parsortix provided a significant association with disease progression (*p* = 0.016) (Table 3). No correlation was shown between response to treatment and phenotypically distinct CTC subsets enriched by any of the methods.

#### 2.4.3. Correlation of CTCs with Survival Measures

**Figure 6:**
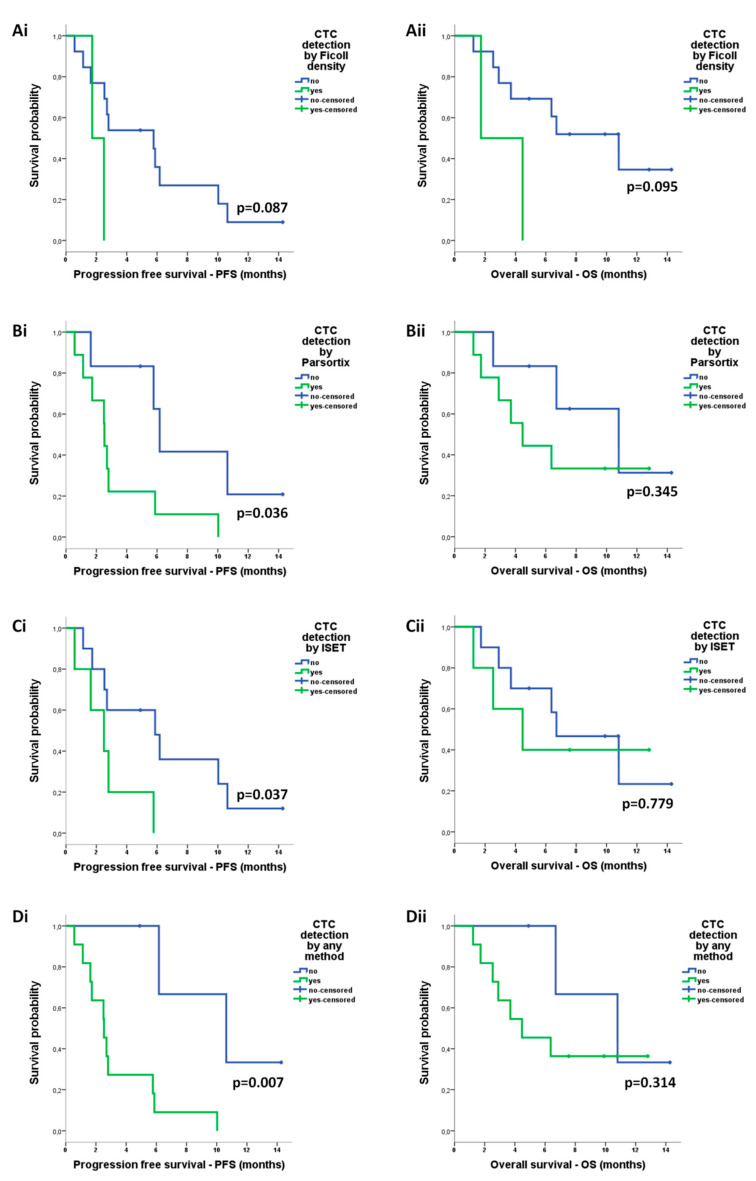
Prognostic relevance of circulating tumor cells (CTCs) enriched by different approaches in patients with non-small cell lung cancer (NSCLC): Kaplan–Meier analysis of progression-free survival (PFS) and overall survival (OS) according to the detection of CTCs after enrichment using (**Ai,ii**) ficoll density, (**Bi,ii**) ISET, (**Ci,ii**) Parsortix, and (**Di,ii**) any method.

The detection of CTCs by either ISET (median: 2.5 vs 5.8 months; *p* = 0.037), Parsortix (median: 2.5 vs 6.2 months; *p* = 0.036) or any method (median: 2.5 vs 10.6 months; *p* = 0.007) was correlated with reduced PFS (Figure 6A–D). CTC detection was not associated with OS (Figure 6A–D).

**Figure 7:**
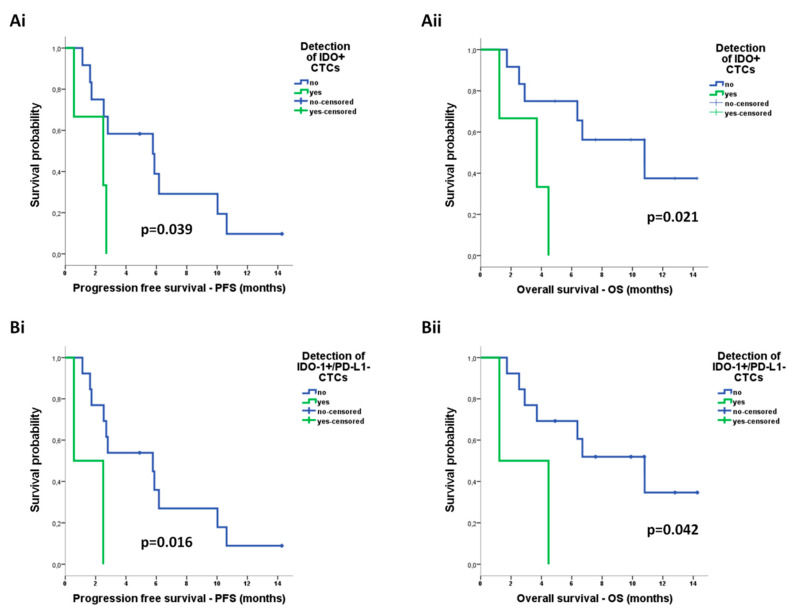
Prognostic relevance of distinct circulating tumor cell (CTC) populations enriched by Parsortix in patients with non-small cell lung cancer (NSCLC): Kaplan–Meier analysis of progression-free survival (PFS) and overall survival (OS) according to the detection of (**Ai,ii**) CTCs positive for indoleamine-2,3-dioxygenase (IDO) expression and (**Bi,ii**) CTCs positive for IDO but negative for programmed cell death ligand-1 (PD-L1) expression (IDO+/PD-L1− phenotype).

Regarding phenotype of CTCs, according to the expression of IDO or PD-L1, there was no correlation between distinct CTC subpopulations detected by ISET and survival measures (Figure S3). Although PD-L1+ CTCs detected by Parsortix were also not predictive of survival outcomes (Figure S3), IDO+ CTCs by Parsortix were associated with shorter PFS (median: 2.5 vs 5.8 months; *p* = 0.039) and shorter OS (median: 3.7 vs 10.8 months; *p* = 0.021) (Figure 7A). Regarding the co-expression phenotypes, IDO+/PD-L1− CTCs detected by Parsortix constituted the only subpopulation associated with reduced PFS (median: 0.6 vs 5.8 months; *p* = 0.016) as well as reduced OS (median: 1.2 vs 10.8 months; *p* = 0.042) (Figure 7B).

Univariate Cox regression analysis revealed an increased risk of relapse for patients harbouring CTCs detected by any system (hazard ratio, HR: 10.733; 95% confidence interval; CI: 1.330–86.579, *p* = 0.026) or CTCs detected by Parsortix (HR: 3.819; 95% CI: 1.005–14.515, *p* = 0.049) and for those harbouring IDO+/PD-L1− CTCs by Parsortix (HR: 6.782; 95% CI: 1.118–41.121, *p* = 0.037) (Table 4). Accordingly, an increased risk of death was recorded for patients harbouring IDO+ CTCs by Parsortix (HR: 5.462; 95% CI: 1.088–27.417, *p* = 0.039) (Table 4). Since the presence and phenotype of CTCs emerged as the only significant parameters in univariate analysis, no multivariate Cox regression analysis was performed.

## 3. Discussion

To promote the role of CTC assessment in the field of personalized cancer care, existing methods for CTC detection should be optimized and their performance should be tested for their compatibility with downstream analyses. In the current report, we evaluated the capture efficiency of different manual and automated CTC detection approaches in patients with NSCLC treated with ICIs as well as the suitability of isolated CTCs for further analyses at the protein expression level. Ficoll density gradient centrifugation, the ISET platform, and the Parsortix system provided the highest enrichment efficacy in spiking experiments, and they were all compatible with protein expression analysis. Subsequent parallel comparison of these methods in patient samples revealed discordant positivity rates and enrichment of phenotypically distinct CTC subsets. The detection of CTCs using either ISET or Parsortix was associated with poor patient outcome, whereas their prognostic value was significantly improved when assessing positivity by any of the three methods. Herein, we also demonstrate for the first time that IDO is expressed on CTCs and that IDO+ CTCs and particularly the IDO+/PD-L1- subset may have significant prognostic relevance in patients with NSCLC treated with anti-PD-1 agents.

In NSCLC, CTC analysis is challenged by the low CTC-positivity rates compared to other cancers [26,27,28]. Different CTC enrichment and detection methods generally vary regarding the yield, purity of samples, release efficiency, and throughput [41]. In the current study, we initially optimized the harvest of cells onto glass slides, which is a crucial step affecting both the recovery and the morphology of cells. We show that direct loading and air-drying of cells is more efficient compared to the cytospin technique, considering the yield, the integrity of cells, and the intensity of the fluorescent staining. In accordance, previous studies reported that the preparation of the cytospins results in significant cell loss and severely impairs cell morphology [46,47,48]. The analytical performance of different manual and automated CTC enrichment methods was then evaluated using the optimized transfer approach.

Among the manual methods tested, ficoll separation alone provided the highest yield compared to ficoll/beads or erythrolysis with or without beads and resulted in the preservation of an intact cell morphology and high intensity of immunofluorescent staining. However, ficoll centrifugation provides samples with high contamination of blood cells. Sample purity affects the throughput and cost of protein expression analysis by defining the number of slides prepared and analyzed through microscopy; however, in contrast to genomic or transcriptomic analysis, the sensitivity or specificity of protein expression analysis may not be affected. Further enrichment by negative selection using magnetic beads significantly improved the purity of samples; however, it resulted in cell loss, worse cell morphology, and reduced staining intensity, corroborating previous evidence showing a negative impact of magnetic beads on downstream fluorescence microscopy readouts [49].

When the two automated systems ISET and Parsortix were compared using spiking experiments, similar recovery rates were recorded for the H1975 and SKMES-1 cell lines, whereas A549 cells were more efficiently enriched by the use of Parsortix. However, in another report, high recovery rates of over 80% were reported for A549 cells by the use of ISET platform [50]. Lower recovery rates compared to ours have been previously reported for A549 cells using Parsortix, possibly attributed to the use of 10-μm gap size cassettes in that study compared to the 6.5-μm gap size used herein [51]. Both ISET and Parsortix systems enriched for morphologically intact cells; however, the downstream expression analysis of ISET-enriched samples was more expensive and time consuming due to the fact that ISET membrane spots were individually stained and analyzed. It should be mentioned though that the entire ISET membrane can be stained and/or immobilized on a single slide; however, this process increases the cost and the time of analysis. These limitations can be overcome by the use of new ISET protocols and consumables, which allow direct membrane sticking on a glass slide as well as the recovery of fixed or unfixed cells from the membrane for downstream analysis [50].

The parallel comparison of ficoll density, ISET, and Parsortix in samples obtained from patients with NSCLC demonstrated that Parsortix results in increased positivity rates and significantly higher CTC counts. However, the direct comparison of ISET and Parsortix revealed only numerical differences, possibly due to the low number of patients analyzed. The Parsortix system uses a microfluidics technology that allows blood flow through a cassette with a stepped structure gradually narrowing in diameter to a critical gap size, thus allowing the capture of unfixed CTCs based on both the size and deformability. We used Parsortix cassettes of 6.5-μm gap size, which demonstrate high efficiency in capturing CTCs from patient samples [45]. On the other hand, the ISET protocol used here allowed the filtration of fixed cells through a membrane consisting of pores of 8 μm in diameter; therefore CTCs of intermediate sizes might have been captured by Parsortix only. It should be noted that previous studies have reported CTC positivity rates of over 75% by using ISET in advanced NSCLC [23,29,33,52] however in these studies, CTC identification was based on cytomorphological criteria assessed by Giemsa staining and/or CD45 negativity. In the current study, instead, CTCs were identified based on CK and/or EpCAM expression, resulting in a detection frequency of 33%. Accordingly, in a study by Hofman et al. [24], CK + CTCs were detected by ISET in 39% of patients with metastatic NSCLC.

Interestingly, despite the significant performance of ficoll density gradient centrifugation in spiking experiments, it resulted in very low positivity rates and CTC counts in patient samples. This is potentially related to the fact that spiking experiments may overestimate the performance of methods since cancer cell lines tend to be more homogenous compared to CTCs, which present significant individual heterogeneity and distinct profiles compared to cancer cell lines [53]. This hypothesis is in line with a recent finding showing a reduction in the cell mass and size of tumor cells undergoing epithelial-to-mesenchymal transition (EMT) [54], a process commonly identified on CTCs [38,39,40].

We interestingly observed that CTC detection using ISET was associated with reduced PFS among patients with NSCLC receiving anti-PD-1 treatment, corroborating previous evidence supporting the prognostic value of CTCs detected by ISET among patients treated with nivolumab [55]. We also demonstrate for the first time that CTC detection using Parsortix is associated with disease progression, reduced PFS, and high risk of relapse among patients treated with anti-PD-1 agents. Importantly, a significant improvement of the prognostic value of CTCs was shown after assessing positivity by any of ficoll density, ISET, and Parsortix isolation methods, suggesting that they may provide complementary clinical information. This observation could be related to the fact that these methods enrich for distinct CTC subpopulations; although CK+/EpCAM+ CTCs was the most prevalent subtype irrespective of the isolation method, CK−/EpCAM+ CTCs were isolated by ficoll and Parsortix only, whereas CK+/EpCAM− cells were detected by ISET only. Our results contrast previous reports demonstrating the detection of EpCAM-negative CTCs by Parsortix [46,51,56]; nevertheless, they highlight the potential heterogeneity of CTCs enriched using different approaches [23,25,57].

PD-L1 expression on CTCs has been previously reported in NSCLC [58,59,60,61,62]. Interestingly, the detection of PD-L1 + CTCs was associated with poor prognosis in patients treated with chemotherapy [60], whereas it was not predictive of survival in those treated with anti-PD-1 agents [55,58]. In accordance, we did not observe any association between PD-L1 + CTCs and patient outcome. Previous reports demonstrated that the persistence [58] or increase [63] of PD-L1 + CTCs after anti-PD-1 treatment was associated with disease progression potentially providing a biomarker for monitoring ICIs efficacy.

IDO expression has been previously described in NSCLC tissue [11,14,64]; however, it is for the first time reported at the CTC level. We demonstrate that IDO + CTCs are identified in patients with NSCLC treated with anti-PD-1 immunotherapy and that their detection by Parsortix is associated with reduced PFS and OS as well with increased risk of death. This finding is in line with the role of IDO in immune evasion and resistance to anti-PD-1 ICIs [8,9,10]. We also demonstrate that IDO is rarely co-expressed with PD-L1 on CTCs of patients with NSCLC, corroborating previous evidence from NSCLC tissue [11,12,14]. These observations indicate that lung cancer cells may preferentially use discrete, nonoverlapping routes to evade antitumor immunity. In accordance, preclinical evidence demonstrates that the combined targeting of IDO and PD-L1 is more effective compared to monotherapy [13], an approach being under investigation in clinical trials [65]. Importantly, we further show that the IDO+/PD-L1− represented the only co-expression phenotype associated with reduced PFS and OS as well as increased risk of relapse.

In the current study, different manual and automated methodologies were compared in spiking experiments using different NSCLC cell lines representative of distinct NSCLC subtypes, considering a variety of quantitative and qualitative parameters. The large volume of peripheral blood collected from healthy blood donors allowed the parallel evaluation of the methods in the same blood sample, which is important considering that critical parameters, such as yield or purity, are donor-dependent [56]. Similarly, methods were evaluated in parallel in blood samples obtained from the same patient, thus allowing the comparison of the methods in real time. In addition, the simultaneous assessment of IDO and PD-L1 expression on CTCs allowed the identification of phenotypically distinct CTC subsets with prognostic significance in patients treated with ICIs, further highlighting the importance of analyzing single CTCs at the protein level. However, image analysis was limited to normal blood cells, and considering that physicochemical properties vary among tumor cells and blood cells [42], we cannot conclude on the integrity or staining intensity of tumor cells based on blood cell analysis. Moreover, the low number of patients included in the current study precludes firm conclusions from being drawn on the relative efficiency of the systems when using clinical samples as well as on the clinical relevance of CTCs and of distinct CTC subsets. Further studies including larger patient cohorts, potentially evaluating the new version of the ISET device, which allows the release of viable, unfixed cells, should be pursued [50].

In summary, the current study aimed to evaluate different methodologies of CTC enrichment considering the yield, the integrity of cell morphology, and the quality of downstream immunofluorescence staining in patients with NSCLC. Herein, we show that ficoll density gradient centrifugation, ISET, and Parsortix result in comparable outcomes in spiking experiments; however, in patient samples, they demonstrate variable efficacy, enrich for distinct CTC subpopulations, and provide complementary prognostic information in NSCLC patients treated with anti-PD-1 ICIs. We also show that IDO, a putative checkpoint of innate and adaptive immunity, is frequently expressed on CTCs and confers adverse prognostic implications in patients treated with anti-PD-1 ICIs. IDO and PD-L1 co-expression is rather uncommon on CTCs, whereas the IDO+/PD-L1− CTC subset is potentially associated with increased prognostic relevance. These observations suggest that this specific immune checkpoint profile may represent an alternative route for immune evasion promoting resistance to anti-PD-1 targeting. Our findings, albeit preliminary, suggest that CTC characterization according to IDO and PD-L1 may promote our understanding of the mechanisms underlying immunotherapy resistance and may provide valuable prognostic information for NSCLC patients treated with anti-PD1 ICIs. Similarly, we recently demonstrated that the parallel assessment of innate (CD47) and adaptive (PD-L1) immune checkpoints on CTCs has significant prognostic and predictive implications in breast cancer [39]. Overall, the above observations highlight the importance of the phenotypic characterization of single CTCs in the effort to obtain a broader view of the systemic antitumor immune response and suggest that CTC analysis in real time might improve the stratification of patients treated with different immunotherapy strategies.

## 4. Materials and Methods

### 4.1. Patients

Peripheral blood (30 mL) was collected from patients with advanced NSCLC (*n* = 15) before the initiation of treatment with anti-PD-1 immune checkpoint inhibitors at the Department of Medical Oncology, University General Hospital of Heraklion, Crete, Greece. Samples were processed within 1 hour for CTC enrichment using different approaches, and enriched samples were further analyzed for the detection and phenotypic characterization of CTCs according to CK, EpCAM, IDO, and PD-L1 expression. For this purpose, two triple immunofluorescence stainings were developed and optimized, CK/EpCAM/CD45 and CK/IDO/PD-L1, and accordingly applied to patient samples. For each patient, a total of 24 slides were analyzed (ficoll slides, *n* = 10; ISET slides, *n* = 10; and Parsortix slides, *n* = 4; and total number, *n* = 360).

Clinical characteristics and follow-up information were prospectively collected. This study was conducted in accordance with the Declaration of Helsinki ethical guidelines and was approved by the Ethics and Scientific Committees of the University General Hospital of Heraklion, Crete, Greece (30/01-11-2017). All patients gave their written informed consent to participate in the study.

### 4.2. Cell Lines

#### 4.2.1. Cell Culture

NSCLC cell lines were obtained from American Type Culture Collection (ATCC, LGC Standards, Wesel, Germany). A549 cells were cultured in high glucose F-12 K (Kaighn’s Modification of Ham’s F-12 Medium) mixture (GIBCO-BRL Co, NY, USA), supplemented with 10% fetal bovine serum (FBS) (GIBCO-BRL) and 1% penicillin/streptomycin (P/S) (GIBCO-BRL). The H1975 cell culture medium was D-MEM 4.5 g/L D-glucose (GIBCO-BRL) with 10% FBS/ 1% P/S. SKMES-1 cells were cultured in MEMα (GIBCO-BRL) medium supplemented with 10% FBS/1% P/S. Cells were maintained in a humidified atmosphere of 5% CO_2_^−^ 95% air at 37 °C, and sub-cultivation was performed using ethylenediaminetetraacetic acid (EDTA)/Trypsin 0.25% (GIBCO-BRL).

Cytospins of H1975 cells and interferon gamma (IFN-γ)-treated A549 cells were also prepared to serve as controls for the optimization of the immunofluorescence stainings. 

#### 4.2.2. Cell Labeling Using CellTracker™ Dye

A549, H1975, and SKMES-1 cells were pre-labeled with the CellTracker™ Green CMFDA dye (Thermo Fisher Scientific, Waltham, MA, USA) prior to spiking experiments in order to facilitate detection and enumeration. Cell culture was at approximately 70–80% confluence on the day of staining. The stock dye solution was dissolved in dimethyl sulfoxide (DMSO) to a final concentration of 6 mM. Cells were washed with phosphate-buffered saline (PBS) and then incubated in 5 mL PBS/CellTracker™ (1:1000, final concentration: 6 μM) for 10 min in a humidified atmosphere of 5% CO_2_^−^ 95% air at 37 °C. Then, 10 mL of standard culture medium including serum was added to quench the reaction and cells were washed with PBS and detached with EDTA/Trypsin 0.25%. In order to confirm that the CellTracker™ Green was taken up by the total of cells, 10 μL of cell suspension was added on a glass slide and analyzed using fluorescence microscopy (Zeiss Axio Imager.A2, Carl Zeiss Microscopy, LLC, New York, NY, USA) in the corresponding fluorescein isothiocyanate (FITC) channel.

### 4.3. Spiking Experiments

Spiking experiments, using representative NSCLC cell lines, were performed to compare the efficacy of different CTC-enrichment methodologies: a) manual (ficoll centrifugation, erythrolysis, and the combination of each one with CD45-magnetic separation) and b) automated (ISET and Parsortix systems). Ficoll centrifugation, the most efficient of the manual methods, was applied along with the automated systems, ISET and Parsortix, in blood samples obtained from patients with advanced NSCLC. Pre-labeled NSCLC cell lines were used in spiking experiments to estimate the yield of the different enrichment and harvest approaches. Non-labeled cells were also used in spiking experiments and were immunofluorescently stained to evaluate the compatibility of the methods with downstream phenotypic analysis. Each spiking experiment was repeated 2–5 times. Recovery rates from replicated experiments are reported as means ± SEM.

To increase the accuracy of the spiking process, pre-labeled tumor cells were suspended in PBS to a final concentration of 20–30 cells/µL and the volume assumed to include 100 cells was added on a glass slide. Labeled cells were recounted under fluorescent microscopy, and if appropriate, the volume of 100 cells was readjusted prior to spiking into the blood samples. The same number of labeled cells was added on 3 glass slides which served as internal controls; the mean value of the counted cells among the 3 control slides represented the final number of spiked cells.

### 4.4. Optimization of Cell Harvest on Glass Slides for Downstream Immunofluorescence Analysis

Pre-labeled H1975 and A549 cells were spiked into PBMCs isolated from peripheral blood (10 mL) of healthy volunteers (100 tumor cells/10^6^ PBMCs). To evaluate the morphology of cells and the intensity of the immunofluorescence staining, non-labeled spiked H1975 and A549 cells were also transferred by different methods and immunofluorescently stained for CK, CD45, and DAPI.

SuperFrost Plus™ adhesion slides (Thermo Fisher Scientific, Waltham, MA, USA) were used in all experiments. First, the impact of coating of the slides with poly-L-lysine (SIGMA-ALDRICH, St. Louis, MO, USA) was estimated by transferring cells on lysine-coated and non-coated slides. Coating was performed by adding slides in a water bath with lysine/water for injection (WFI) (1:10) for 1 h and by washing them twice with WFI and air-drying at room temperature (RT).

Three different harvest approaches were also tested: a) 2 min cytospin at 200 g, b) 5 min cytospin at 500 g, and c) direct cell loading on slides and air-drying at RT.

### 4.5. CTC-Enrichment Methodologies

Peripheral blood (30 mL) was obtained from healthy volunteers and spiked with pre-labeled A549, H1975, and SKMES-1 cells. Samples of 5 mL blood spiked with 100 tumor cells (20 cells/mL blood) were processed by each enrichment approach. Non-labeled tumor cells were also stained for CK/CD45/DAPI in order to evaluate the compatibility of different enrichment methodologies with downstream phenotypic analysis.

The following manual enrichment methods were tested: a) ficoll-density gradient centrifugation, b) red blood cell lysis (erythrolysis), c) combination of ficoll with negative magnetic separation, and d) combination of erythrolysis with negative magnetic separation. The automated ISET and Parsortix systems were also evaluated. 

#### 4.5.1. Ficoll Density Gradient Centrifugation

Peripheral blood mononuclear cells (PBMCs) were isolated by Ficoll-Hypaque density gradient centrifugation (d = 1.077 g/mL) (Merck KGaA, Darmstadt, Germany) at 650 × *g* for 30 min. PBMCs were washed twice with PBS, and aliquots of 1 × 10^6^ cells were directly loaded and air-dried on lysine-coated glass slides.

#### 4.5.2. Red Blood Cell Lysis—Erythrolysis

Blood samples were diluted 1: 9 with red blood cell lysis buffer (0.8% NH_4_Cl, 0.1%KHCO_3_, 0.1 mM EDTA, pH 7.4) and agitated for 20 min in dark at RT. Following centrifugation at 500 g for 10 min at 21 °C, cells were washed twice with cold wash buffer (PBS/0.1% FBS/2 mM EDTA) by centrifugation at 500 g for 5 min at 4 °C. Aliquots of 1 × 10^6^ cells were loaded and air-dried on lysine-coated glass slides.

#### 4.5.3. Magnetic Separation Using CD45 Dynabeads 

Following enrichment of cells by ficoll separation or erythrolysis, negative magnetic separation was additionally performed using magnetic beads coated with anti-CD45 antibody (Invitrogen, Carlsbad, CA, USA), according to the manufacturer’s instructions. In brief, beads were washed with Buffer 1 (PBS/0.1% FBS/2 mM EDTA) and then added to the ficoll- or erythrolysis-enriched cells at a concentration of 100 µL of beads per 10 × 10^6^ of cells suspended in 1 mL of buffer 1. Following incubation for 30 min at 4 °C with rotation, tubes were placed in a magnet for 10 min. The supernatant (including tumor cells) was transferred in FBS pre-coated tubes and washed with cold buffer 1 at 600 × *g* for 15 min at 4 °C. Cells were loaded and air-dried on lysine-coated glass slides.

#### 4.5.4. ISET Filtration

Tumor cells were enriched using the size-based ISET platform (Rarecells, Diagnostics, Paris, France). Briefly, blood samples were diluted in 1:10 erythrocyte-lysis buffer (Rarecells, Paris, France) and incubated for 10 min at room temperature (RT). Samples were filtrated through the ISET membranes, bearing pores of 8 μm in diameter at −10 kPa pressure. Following filtration, membranes were washed with PBS and WFI, air-dried at RT, and stored at −20 °C. The membrane spots (*n* = 10), each one corresponding to 1 mL whole blood, were individually cut out and immobilized on 10 glass slides using adhesive ribbon to be further stained and analyzed by microscopy, as previously described by Pailler et al. [33,34,35,36,37,38,39,40,41,42,43,44,45,46,47,48,49,50,51,52,53,54,55,56,57,58,59,60,61,62,63,64,65,66]. Although the entire ISET membrane can be stained and/or immobilized on a single slide, we did not select this procedure since it required larger volumes of antibody solutions compared to the staining of individual spots (a minimum of 2 mL vs 100 μL per spot to a final volume of 1 mL, respectively). Furthermore, the immobilization of more than one spot per slide increased the technical difficulty in the evaluation of a microscopically non-flat membrane, thus increasing the time required for screening. These limitations could be overcome by using a different isolation protocol [50], which may allow the harvest of cells and consequently their transfer on a single slide.

#### 4.5.5. Parsortix Separation

Tumor cells were enriched by the size- and deformability-based Parsortix system (Angle plc, Guildford, UK). Blood separation was performed using cassettes with a gap size of 6.5 μm and a separation pressure of 99 mbar, as proposed by other studies [45,67]. Cells captured in the cassette were harvested, loaded on lysine-coated glass slides, and air-dried. Harvested cells on slides, rather than captured cells in the cassette, were counted in order to estimate the overall recovery of the method. Accordingly, the staining and evaluation of patient samples was performed on cells harvested on slides rather than in the cassette.

### 4.6. Immunofluorescence Approaches for CTC Detection and Characterization

#### 4.6.1. CK/EpCAM/CD45 Staining

The triple CK/EpCAM/CD45 staining was developed and optimized using ficoll-enriched H1975 spiked cells, as controls. For this purpose, positive controls (including all three primary and the corresponding secondary antibodies) and negative controls, one for each marker (including the secondary Immunoglobulin G (IgG) isotype antibody only and omitting the corresponding primary antibody) were prepared as previously described [38,39].

The optimized staining protocol included a fixation step with PBS/FA 3.7% for 15 min, RT; permeabilization with PBS/Triton X-100 0.1% for 10 min, RT; and blocking with PBS/FBS 5% for 1 h, RT. The primary antibodies, mouse anti-EpCAM (1:1000) (Clone VU-1D9, Novus Biologicals, LLC, Centennial, CO, USA; NBP2-33078) and rabbit anti-CD45 (1:100) (H-230; Santa Cruz Biotechnology, Inc. Dallas, Texas, USA; sc-25590), were incubated for 1 h, RT. The corresponding secondary antibodies, Alexa Fluor 555 anti-mouse (1:600) and Alexa Fluor 633 anti-rabbit (1:1000) (Thermo Fisher Scientific, Waltham, MA, USA) were incubated for 45 min, RT. CKs were detected by two different Alexa Fluor 488-conjugated clones: mouse AE1/AE3 (1:100) (Thermo Fisher Scientific, Waltham, MA, USA) and mouse C11 (1:200) (Novus Biologicals, LLC, Centennial, CO, USA) after overnight incubation at 4 °C. DAPI antifade (Invitrogen, Carlsbad, CA, USA) was finally added to identify cell nuclei.

Accordingly, the CK/EpCAM/CD45 staining was evaluated in ISET- and Parsortix- enriched H1975 cells. No difference was observed regarding the specificity, intensity, or localization of the three markers among the differentially processed samples; therefore, ficoll-prepared slides were selected to serve as internal controls during the staining of patient samples.

#### 4.6.2. CK/IDO/PD-L1 Staining

A549 cells were treated with IFN-γ (Peprotech EC Ltd, London, UK) for the induction of IDO expression, according to the manufacturer’s instructions. For this purpose, A549 cells were plated at a density of 4 × 10^5^ cells in 6 well plates, allowed to adhere, and then treated with different concentrations of IFN-γ (10, 25, 50, 100, and 500 ng/mL) for 24 h and 48 h. Cells were detached with 0.25% Trypsin/EDTA, and cytospins of 5 × 10^5^ cells were prepared in order to serve as controls for the optimization of the CK/IDO/PD-L1 staining.

The optimum induction of IDO expression in A549 cells was obtained after IFN-γ treatment at 100 ng/mL for 48 h (Figure S4). Accordingly, cytospins of IFN-γ-treated A549 cells were used for the optimization of the triple CK/IDO/PD-L1 staining by the use of positive and negative controls for each marker, as described above. These slides also served as controls for patient samples (Figure S5).

The optimized staining protocol included fixation with PBS/FA 3.7% for 15 min, RT (excluding the ISET samples, as described above); permeabilization with PBS/Triton X-100 0.1% for 10 min, RT; and blocking with PBS/FBS 5% for 1 h, RT. The primary antibodies, mouse anti-IDO (1:150) (Clone 1F8.2, Merck, Darmstadt, Germany, MAB10009) and rabbit anti-PD-L1 (1:100) (Clone E1L3N, Cell Signaling, Danvers, MA, USA, #13684), were incubated for 1 h, RT. The corresponding secondary antibodies, Alexa Fluor 555 anti-mouse (1:600) and Alexa Fluor 633 anti-rabbit (1:800) were incubated for 45 min, RT. CKs were detected by two different Alexa Fluor 488-conjugated clones: mouse AE1/AE3 (1:100) (Thermo Fisher Scientific, Waltham, MA, USA) and mouse C11 (1:200) (Novus Biologicals, LLC, Centennial, CO, USA) after overnight incubation at 4 °C. DAPI antifade (Invitrogen, Carlsbad, CA, USA) was added for the cell nuclei staining.

### 4.7. Enrichment, Detection, and Phenotypic Characterization of CTCs from Patients’ Samples

Peripheral blood (30 mL) was obtained at the middle of vein puncture and collected in EDTA tubes (Becton Dickinson, Franklin Lakes, NJ, USA) after the first 5 mL was discarded to avoid contamination with epithelial skin cells. CTCs were in parallel enriched by ficoll density gradient centrifugation, ISET and Parsortix (10 mL blood each), and the following samples were accordingly prepared per patient: a) ~10 ficoll slides (depending on the number of total PBMCs; 1 × 10^6^ PBMCs per slide), b) 10 ISET slides (1 spot per slide, corresponding to 1 mL blood), and c) 4 Parsortix slides (1 slide corresponds to 2.5 mL blood) (total number of slides: *n* = 360).

Samples were divided in two and were individually stained for CK/EpCAM/CD45 and CK/IDO/PD-L1. The detection and characterization of CTCs were performed using the Ariol microscopy system Genetix, New Milton, UK) as previously described [37,38,39], by two observers (A.I.S. and C.V.) who were blinded to each other’s findings and patients’ clinical data.

Among the samples stained for CK/EpCAM/CD45, two individual screenings for the detection of CK and EpCAM expression were performed. CD45 expression served as an exclusion marker. As described above, spiked H1975 cell cytospins were included in all immunofluorescence stainings performed in patient samples in order to define the positivity and negativity of each marker. The detection of at least one intact, nucleated cell, positive for CK and/or EpCAM, was used to define CTC positivity, as previously described [38,39,57].

Accordingly, samples stained for CK/IDO/PD-L1 were screened for CTCs based on CK expression, and CK + CTCs were subsequently characterized according to the expression of IDO and PD-L1. The detection of at least 1 CTC of a specific phenotype was used to define positivity for the respective phenotype, as previously described [37,38,39].

### 4.8. Image Analysis

Representative Ariol microscopy images from samples processed by different harvest and enrichment approaches were obtained at the same exposure and magnification (400×). ImageJ software (1.52t version, NIH) was used to analyze parameters associated with cell morphology, such as nuclear area and roundness, as well as the intensity of the fluorescent staining. Due to the limited number of tumor cells included in slides that were immunofluorescently stained during the spiking experiments, the image analysis was limited to normal blood cells only. Thus, whenever we refer to nuclear area, roundness, and staining intensity, we refer to normal blood cells. Fluorescence intensity was expressed as Corrected Total Cell Fluorescence (CTCF), as previously described [68]. 

### 4.9. Statistical Analysis

Recovery rates from replicated spiking experiments are presented as means ± standard error of mean (SEM). T-test and one-way ANOVA test were used to compare the recovery between two or more than two methodologies, respectively. Mann–Whitney t-test was used to compare the parameters assessed by ImageJ analysis between two different methods. Fisher’s exact test was used to investigate possible correlations of CTCs and distinct cell subsets with patient and disease characteristics. Kaplan–Meier analysis was used to estimate survival curves. Progression-free survival (PFS) was calculated from the start of immunotherapy until disease progression or death from any cause. Overall Survival (OS) was calculated from the immunotherapy initiation until death from any cause. Univariate Cox regression analysis was performed to investigate the associations between different parameters and the risk for relapse and death. Statistical analyses were performed using IBM SPSS Statistics version 20. *p*-values were calculated by two-sided tests and were considered statistically significant at the *p* < 0.05 and *p* < 0.001 level. 

## 5. Conclusions

In the current study, a series of manual and automated CTC-enrichment approaches were compared for their efficiency to enrich CTCs and for their compatibility for downstream protein expression analysis in NSCLC. The results presented herein show that different CTC enrichment methods provide discordant positivity rates and CTC counts and enrich for distinct CTC populations. CTC detection is associated with adverse outcomes in NSCLC patients treated with ICIs. Further phenotypic analysis of CTCs according to IDO and PDL1 checkpoints showed that IDO+ CTCs and particularly the IDO+/PD-L1− subset are associated with poor clinical outcomes in patients treated with ICIs. Although these findings merit further validation in larger patient cohorts, they suggest that the detection and characterization of CTCs for putative immune checkpoints is feasible in NSCLC and may provide promising prognostic and/or predictive biomarkers for monitoring ICI efficacy. 

## Figures and Tables

**Table 1:** Frequency of CTC detection in patients with non-small cell lung cancer (NSCLC) using manual and automated approaches.

Enrichment Method	CTC Detection ^a^			Friedman Exact Test (Mean Ranks)
	Positive Patients no (%)	Total CTCs no	No. of CTCs/Patient Mean (range)	
Ficoll	2 (13)	3	0.2 (0–2)	1.67
ISET	5 (33)	8	0.53 (0–3)	1.87
Parsortix	9 (60)	32	2.13 (0–12)	2.47
Any method	11 (73)	43	2.87 (0–12)	*p* = 0.010 *

^a^ CTCs detected by any staining process; * Friedman paired exact test; statistical significance at the p < 0.05 level.

**Table 2:** Characteristics of patients with non-small cell lung cancer (NSCLC).

Patients	n (%)	Patients	n (%)
Age, years		Metastatic sites	
median (range)	70 (61–82)	Lung	12 (80)
Gender		Liver	4 (26.7)
Male	13 (86.7)	Brain	2 (13.3)
Female	2 (13.3)	Bones	5 (33.3)
Smoking status		Adrenal gland	2 (13.3)
Ex-smoker	4 (26.7)	Lymph nodes	10 (66.7)
Current smoker	11 (73.3)	NLR	
Performance status (ECOG)		<3	3 (20)
		≥3	12 (80)
0–1	9 (60)	Line of treatment	
2	6 (40)	2nd	10 (66.7)
Histology subtype		3rd	5 (33.3)
Squamous	10 (66.7)	Best response to immunotherapy	
Non-squamous	5 (33.3)	Partial response	3 (20)
No. of organs affected		Stable disease	3 (20)
1–2	10 (66.7)	Progressive disease	8 (53.3)
≥3	5 (33.3)	Non-evaluable ^a^	1 (6.7)

ECOG: Eastern Cooperative Oncology Group; NLR: neutrophil–lymphocyte ratio; ^a^ Treatment terminated due to toxicity.

**Table 3:** CTC detection using different approaches according to best response to anti-programmed cell death-1 (PD-1) immunotherapy.

Best Response	CTC-Positive Patients, *n* (*%*)			
	Ficoll	ISET	Parsortix	Any Method
PR	0 (0)	0 (0)	0 (0)	0 (0)
SD	0 (0)	1 (33.3)	1 (33.3)	2 (66.7)
PD	2 (25)	3 (37.5)	7 (87.5)	8 (100)
*p*-value	1.000	0.748	0.016*	0.006 *

PR: partial response (*n* = 3), SD: stable disease (*n* = 3), PD: disease progression (*n* = 8) * Statistical significance at the p < 0.05 level; Fisher’s exact test, two tailed.

**Table 4:** Univariate Cox-regression analysis for PFS and OS among patients with NSCLC.

Univariate Cox-Regression Analysis	Progression Free Survival (PFS)		Overall Survival (OS)	
Covariates	HR (95% CI)	*p*-Value	HR (95% CI)	*p*-Value
Age (<70 vs ≥70 years)	1.745 (0.518–5.880)	0.369	3.282 (0.750–14.360)	0.115
Gender (male vs female)	2.329 (0.297–18.293)	0.421	1.293 (0.159–10.550)	0.810
Smoking status (current vs ex-smoker)	1.279 (0.379–4.318)	0.691	0.747 (0.186–3.004)	0.682
Performance status (2 vs 0–1)	1.022 (0.329–3.174)	0.970	1.875 (0.502–7.011)	0.350
Histology (squamous vs adenocarcinoma)	2.364 (0.646–8.655)	0.194	2.800 (0.566–13.843)	0.207
Metastatic sites (yes vs no)				
Lung	1.716 (0.369–7.969)	0.491	3.433 (0.391–30.173)	0.266
Liver	1.928 (0.562–6.614)	0.297	1.375 (0.342–5.524)	0.654
Brain	0.429 (0.055–3.373)	0.421	0.773(0.095–6.309)	0.810
Bones	1.295 (0.414–4.051)	0.656	2.720 (0.726–10.188)	0.138
Adrenal gland	0.022 (0.000–5.625)	0.177	0.340 (0.037–3.094)	0.338
Lymph nodes	1.588 (0.485–5.199)	0.445	1.567 (0.382–6.434)	0.533
No of organs affected (≥3 vs <3)	1.330 (0.425–4.160)	0.624	3.063 (0.820–14.443)	0.096
Neutrophil–lymphocyte ratio (≥3 vs <3)	4.400 (0.562–34.432)	0.158	2.607 (0.323–21.021)	0.368
Line of treatment (2nd vs 3rd)	2.089 (0.567–17.697)	0.268	4.367 (0.544–35.044)	0.165
CTC detection (yes vs. no)				
Ficoll-positive	4.237 (0.700–25.653)	0.116	4.351 (0.787–24.066)	0.092
ISET-positive	3.746 (0.994–14.117)	0.051	1.220 (0.304–4.892)	0.779
Parsortix-positive	3.819 (1.005–14.515)	0.049*	1.946 (0.478–7.921)	0.353
Positive by any method	10.733 (1.330–86.579)	0.026 *	2.249(0.448–11.288)	0.325
CTC subsets detected by Parsortix (yes vs. no)				
IDO + CTCs	4.305 (0.951–19.492)	0.058	5.462 (1.088–27.417)	0.039 *
IDO+/PD-L1− CTCs	6.782 (1.118–41.121)	0.037 *	4.964 (0.900–27.376)	0.066

NSCLC: non-small cell lung cancer; HR: hazard ratio; CI: confidence interval; CTC: circulating tumor cell; IDO: indoleamine-2,3-dioxygenase; PD-L1: programmed cell death ligand-1. * Statistical significance at the *p* < 0.05 level.

## Supplementary Materials

The following are available online at https://www.mdpi.com/2072-6694/12/6/1556/s1, Figure S1: Comparison of cell morphology and staining intensity among cells processed by different harvest and enrichment approaches: ImageJ analysis was used to compare the nuclear area, roundness, and the intensity of CD45 fluorescent signal in normal blood cells. Two-sided Mann–Whitney t-test; statistical significance at the *p* < 0.001 level. AU: arbitrary units; CTCF: Corrected Total Cell Fluorescence, Figure S2: Representative image of a spiked SKMES-1 cell captured on an ISET membrane pore (Ariol microscopy system, 400×), Figure S3: Survival analysis based on distinct CTC subpopulations in patients with NSCLC: Kaplan–Meier plots for progression-free survival (PFS) and overall survival (OS) according to the detection of Ai–ii) IDO+ CTCs by ISET, Bi–ii) PD-L1+ CTCs by ISET, and Biii–iv) PD-L1+ CTCs by Parsortix, Figure S4: Induction of IDO expression in A549 cell line by treatment with IFΝ-γ. Representative images of IDO expression (orange) and nuclei DAPI staining (blue) in A549 cells upon IFΝ-γ treatment at different concentrations and incubation times; Ariol microscopy system (×100), Figure S5: Optimized staining of CK/IDO/PD-L1 among IFΝ-γ-treated A549 control cells: Representative image of nuclei DAPI staining (blue), CK (green), IDO (orange), and PD-L1 (red) in the triple-positive control slide; Ariol microscopy system (100×), Table S1: Throughput of manual and automated enrichment approaches in spiking experiments.
