# Supplementary material for: Optimization of the Enrichment of Circulating Tumor Cells for Downstream Phenotypic Analysis in Patients with Non-Small Cell Lung Cancer Treated with Anti-PD-1 Immunotherapy
Source: Cancers (Basel). 2020 Jun 12;12(6):1556. doi: 10.3390/cancers12061556 (PMC7352396; doi:10.3390/cancers12061556)
Supplement: Supplementary file 1 [file cancers-12-01556-s001.pdf]

Supplementary Materials

Table S1. Throughput of manual and automated enrichment approaches in spiking experiments.

**Enrichment Method**	**Enrichment Duration**	**Contaminated Cells** *Mean Number (range)*	**Slides** *Mean Number*
Ficoll	1 h 30 min	8.9×10^6 ($7–11 \times 10^6$)	9
Ficoll & beads	2 h 25 min	0.8×10^6 ($0.5–1.2 \times 10^6$)	1
Erythrolysis	45 min	13.7×10^6 ($10–22.3 \times 10^6$)	14
Erythrolysis & beads	2 h	2.1×10^6 ($1–2.5 \times 10^6$)	2
ISET	30 min	9×10^4 ($5.7–19.4\times 10^4$)	10
Parsortix	1 h 30 min	4.6×10^3 ($1.6–9.7 \times 10^3$)	1

The duration time and counts of cells and slides, correspond to the processing of blood samples of 5mL.

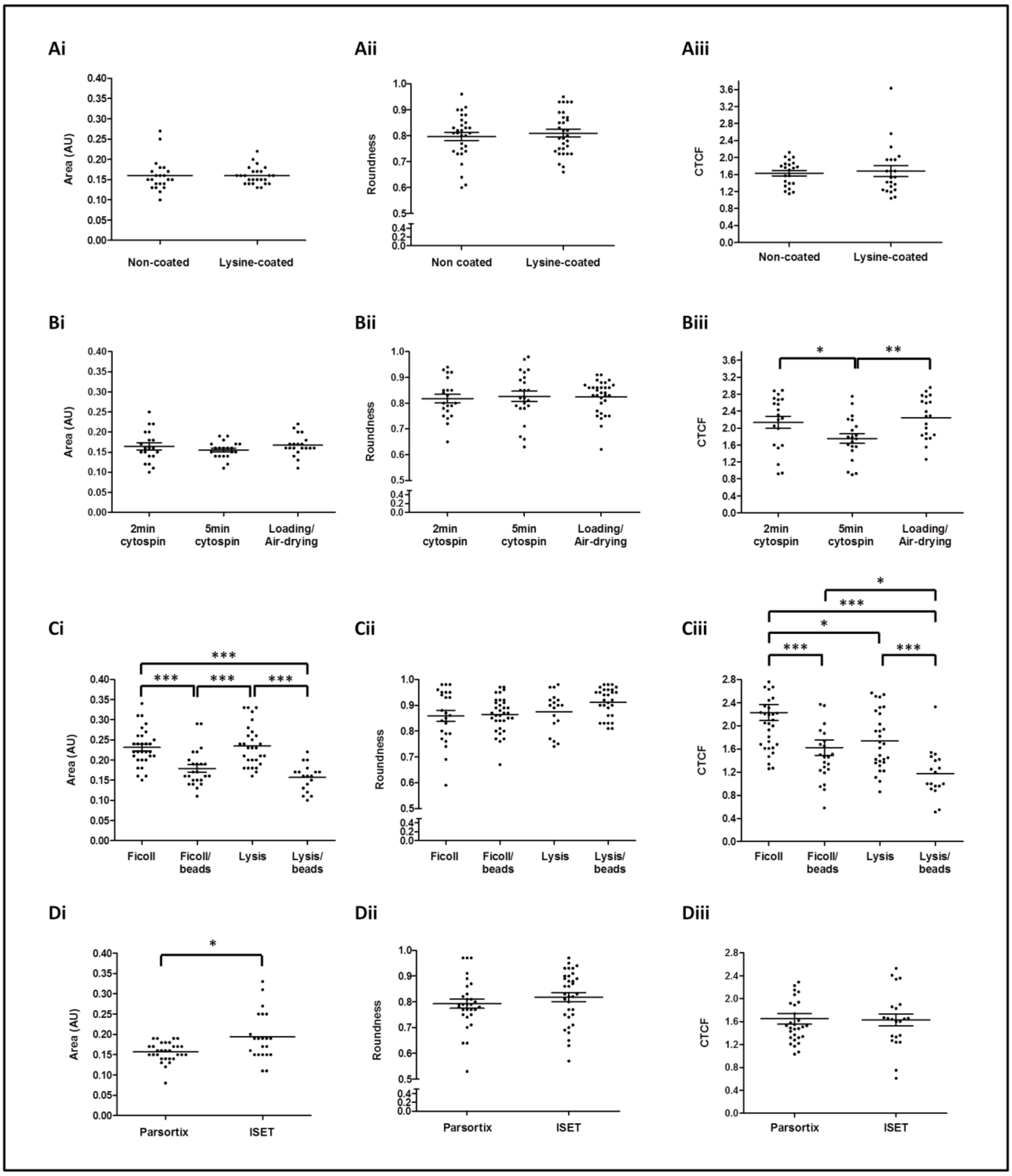

Figure S1. Comparison of cell morphology and staining intensity among cells processed by different harvest and enrichment approaches. ImageJ analysis was used to compare the nuclear area, nuclear roundness, and the intensity of CD45 fluorescent signal in normal blood cells. Two-sided Mann-Whitney t-test; statistical significance at the $p < 0.001$ level. AU: arbitrary units; CTCF: Corrected Total Cell Fluorescence.

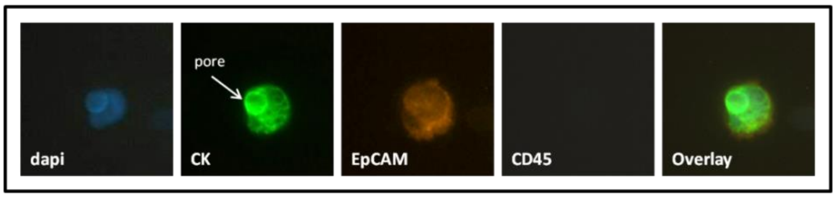

Figure S2. Representative image of a spiked SKMES-1 cell captured on an ISET membrane pore (Ariol microscopy system, 400x).

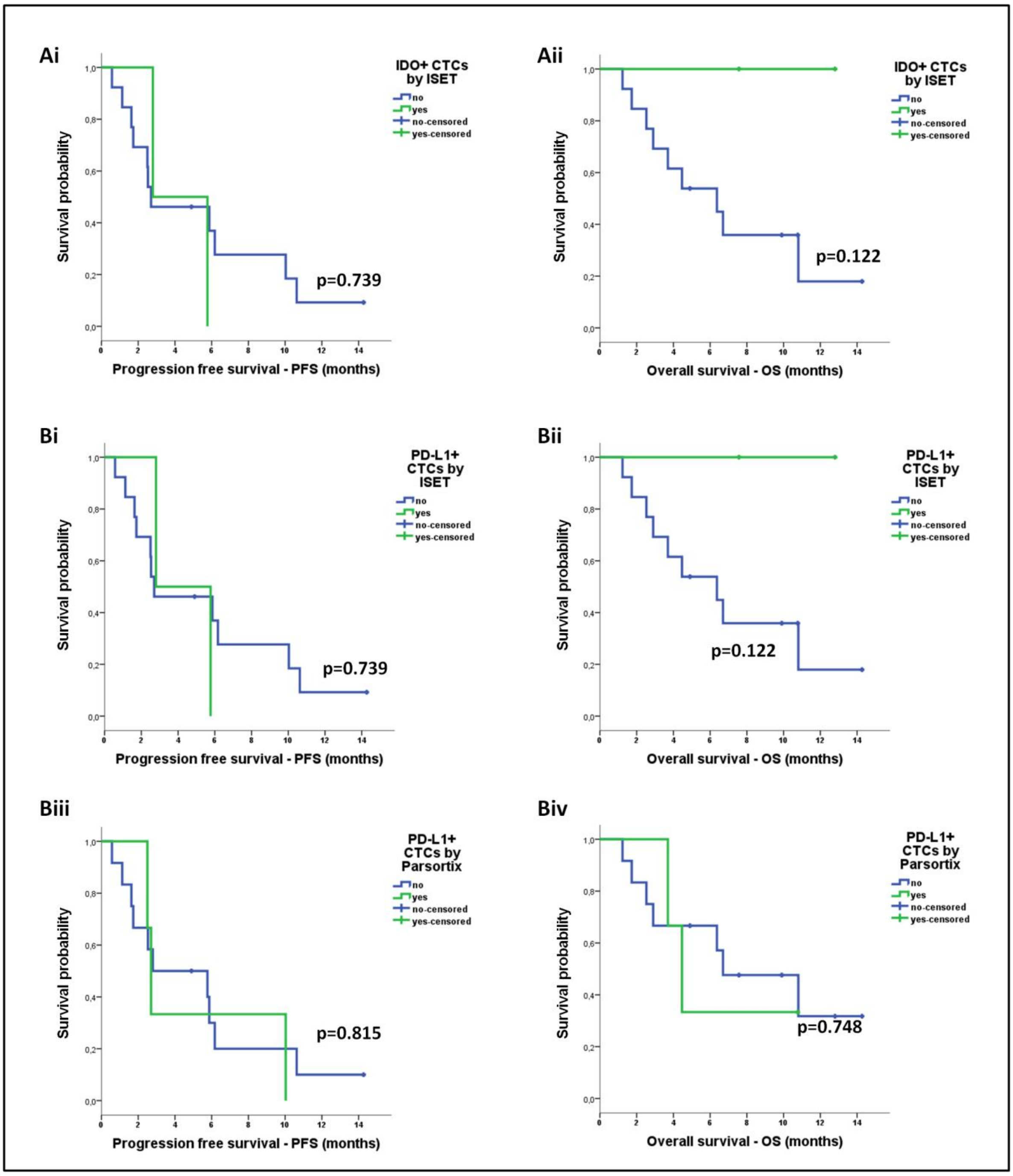

Figure S3. Survival analysis based on distinct CTC subpopulations in patients with NSCLC. Kaplan-Meier plots for progression-free survival (PFS) and overall survival (OS) according to the detection of (**Ai-ii)** IDO+ CTCs by ISET, (**Bi-ii)** PD-L1+ CTCs by ISET, and (**Biii-iv)** PD-L1+ CTCs by Parsortix.

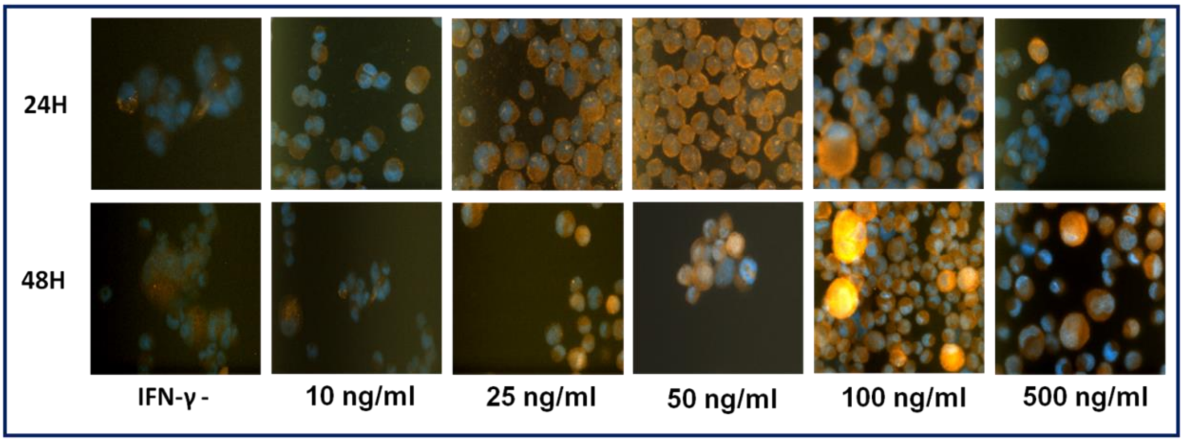

Figure S4. Induction of IDO expression in A549 cell line by treatment with IFN-γ. Representative images of IDO expression (orange) and nuclei dapi staining (blue) in A549 cells upon IFN-γ treatment at different concentrations and incubation times; Ariol microscopy system (x100).

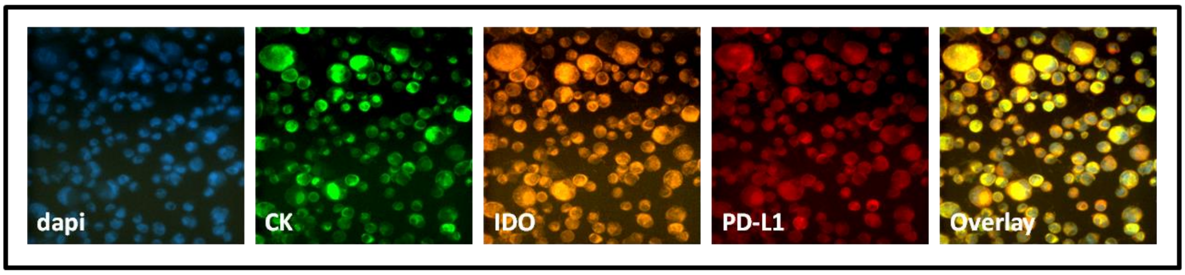

Figure S5. Optimized staining of CK/IDO/PD-L1 among IFN-γ-treated A549 control cells. Representative image of nuclei dapi staining (blue), CK (green), IDO (orange), and PD-L1 (red) in the triple-positive control slide; Ariol microscopy system (x100).
